# Common Superficial Bacterial Skin Infections Self-Reported by 1047 Greek Competitive Swimmers: A 2021 Retrospective Study

**DOI:** 10.3390/idr17050133

**Published:** 2025-10-20

**Authors:** Eleni Sfyri, Niki Tertipi, Vasiliki Kefala, Vasiliki-Sofia Grech, Efstathios Rallis

**Affiliations:** Department of Biomedical Sciences, School of Health and Care Sciences, University of West Attica, 12243 Athens, Greece; ntertipi@uniwa.gr (N.T.); valiakef@uniwa.gr (V.K.); vgkrek@uniwa.gr (V.-S.G.); erallis@uniwa.gr (E.R.)

**Keywords:** cutaneous bacterial infections, competitive swimmers, folliculitis, pitted keratolysis, impetigo, Greece

## Abstract

Background/Objectives: Superficial bacterial skin infections are common, particularly among athletes. In swimming, data on folliculitis, impetigo, and pitted keratolysis are limited. This study aimed to evaluate the prevalence of superficial bacterial skin infections in young competitive swimmers from Greek clubs. Methods: An anonymous questionnaire was distributed to all swimming clubs through the Hellenic Swimming Federation, with a request to forward it to their members. It was completed by 1047 swimmers or their parents. Data collection included skin conditions along with explanatory notes, as well as additional information such as gender, swimming age category, season of occurrence, training routine details, and hygiene-related behaviors. Results: The study showed that 2.7% of participants reported being affected by folliculitis, 10.9% by impetigo, and 3.2% by pitted keratolysis. Infections were significantly correlated with age categories. Folliculitis and pitted keratolysis were less frequent and were mainly reported by adolescent and adult swimmers. In contrast, impetigo was more common and primarily affected younger age groups. Specific behaviors—such as placing towels and clothes on locker room benches—were significantly associated with pitted keratolysis (*p* = 0.036) and impetigo (*p* < 0.001). Sharing equipment was associated with all three infections. Conclusions: This study highlights the high prevalence of bacterial skin infections in Greek swimmers, likely due to moisture exposure, shared equipment, and specific hygiene habits.

## 1. Introduction

Skin infections constitute a common clinical condition affecting both the general population and athletes [1]. Notably, swimming has been identified as a factor conferring an increased susceptibility to these infections [2]. These infections are influenced by factors such as moisture, temperature changes, chemical exposure, and sunlight, and are often linked to swim gear and swimmer habits [3,4,5,6]. Superficial bacterial skin infections, which occur in the general population, are among the types of skin infections that can negatively affect swimmers’ training and competitive performance.

Folliculitis is a common, typically mild inflammation of hair follicles, presenting with red papules or pustules. It can be caused by bacterial, fungal, viral, or parasitic infections, as well as chemical or physical irritants [7,8]. Risk factors include diabetes, obesity, frequent shaving, prolonged antibiotic use, immunosuppression, and exposure to communal water facilities [9]. Hot tub folliculitis, often affecting shaved or covered areas, appears within 8–48 h after water exposure and accounts for 80% of water-related cases, with the remaining 20% linked to swimming pools [10]. It can present in any area of the body but is often found in occluded areas, is frequently painful, and mildly pruritic [8]. Studies report high incidence among swimmers and winter athletes, with one outbreak affecting 590 individuals at a Utah water park [10].

Impetigo is a highly contagious skin infection affecting the upper epidermis, primarily caused by Gram-positive bacteria. It spreads through direct contact with infected individuals, contaminated surfaces, or via self-inoculation. There are two main forms: bullous and non-bullous [11]. The non-bullous type is most common. This infection can appear in areas such as the nose, perineum, axillae, and fingers, often following skin injuries like insect bites, cuts, burns, herpes lesions, or atopic dermatitis. Impetigo occurs frequently in children of all sexes and ethnicities, especially in warm, humid climates during summer and fall [12]. It is also common in athletes involved in contact sports or those who share equipment [10]. Participation in seawater activities is associated with an increased risk, particularly in children [13].

Pitted keratolysis is a common bacterial infection of the soles, primarily caused by Gram-positive bacteria such as *Micrococcus* and *Corynebacterium* [14]. It affects individuals who wear occlusive footwear for long periods—such as soldiers, athletes, and marine workers—as well as those who walk barefoot [14]. Clinically, it presents with small pits (1–3 mm) and erosions, often accompanied by a strong malodor [10,15]. It occurs at all ages but is more frequent in adolescents and young adults, particularly males, due to more frequent use of closed footwear [15]. Treatment is effective and focuses on reducing moisture to manage and prevent recurrence.

This study aims to assess the prevalence of superficial bacterial skin infections among Greek competitive swimmers. The existing literature is limited, with most research focusing on children, the general population, or athletes in other sports disciplines [15]. The central hypothesis is that frequent participation in competitive swimming, combined with hygiene practices and shared equipment use, may increase infection risk. The lack of relevant epidemiological data in Greece highlights a gap in the literature. This study addresses that gap by providing context-specific findings that can support the development of targeted, evidence-based prevention strategies to minimize training disruptions and performance impacts.

## 2. Materials and Methods

This cross-sectional study aimed to assess the prevalence of superficial bacterial skin infections among competitive swimmers in Greece. Ethical approvals were secured from the University of West Attica (52645 20 July 2020) and the Hellenic Swimming Federation (787 15 March 2019). An online survey was conducted from June to December 2021. The study employed a self-selected sampling method, which was the only feasible approach given the challenges in reaching the entire population of competitive swimmers in Greece.

Participants were drawn from swimming clubs across the country. Among 182 clubs contacted, swimmers from 80 clubs participated. Data collection was conducted in two phases:

- In Phase A, the Hellenic Swimming Federation sent the questionnaire via email to 143 registered swimming clubs. The remaining 39 clubs, which could not be reached by email due to lack of contact information, were informed through social media. Swimmers who responded in Phase A indicated their club affiliation in the questionnaire (Supplementary S1).
- In Phase B, the researchers contacted coaches and club managers from clubs that had not responded in Phase A, encouraging them to redistribute the questionnaire to their athletes (Supplementary S1). A total of 60 clubs were selected based on the possibility of telephone contact, and swimmers from 33 of these clubs responded during this Phase.

**Figure 1:**
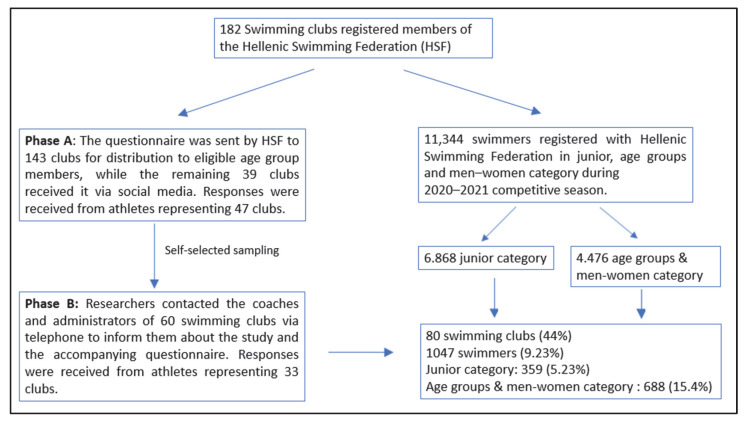
Flowchart of Participant Recruitment and Response.

The total number of questionnaire recipients was 11,344 swimmers or their parents, who were registered members of the Hellenic Swimming Federation during the 2020–2021 competitive season. A total of 1047 competitive swimmers participated, representing the Junior category (ages 9–12), the Age Group categories (ages 13–18), which include the subcategories 13–14, 15–16, and 17–18 years of age, as well as the Men–Women category. This sample size of 9.23% was deemed statistically adequate and representative of the overall swimmer population [16]. Participation was voluntary and anonymous (Figure 1).

This study is part of a broader project examining the prevalence and characteristics of dermatological conditions in swimmers. To ensure scientific rigor, the questionnaire underwent test–retest reliability testing and assessment of content and face validity [17]. The test–retest procedure involved administering the same questionnaire to 57 swimmers or their parents, members of a swimming club, twice within a 15-day interval. The questionnaire included closed-ended questions, either dichotomous (YES/NO) or multiple-choice, where multiple answers were allowed.

During the first administration, an open-ended item invited participants to share comments about specific questions, focusing on clarity, relevance, and wording. These responses led to clarifying edits, mainly regarding the skin conditions mentioned. In the second administration, a revised version of the questionnaire was used, primarily incorporating clarifying explanations for the listed skin conditions.

Cohen’s Kappa (κ) was used to assess agreement between the two administrations, suitable for categorical variables. Each response option in multiple-choice questions was coded as a binary variable. The Kappa value of 0.75 indicated good reliability, influenced by improvements in question clarity based on participant feedback.

Content validity was evaluated prior to retesting by a dermatologist and a methodology expert, both of whom confirmed the questionnaire’s appropriateness. Minor wording adjustments were made accordingly. These procedures ensured that the instrument was both methodologically reliable and clinically relevant for studying dermatological conditions in the swimming population.

For the main study, the questionnaire was distributed via Google Forms and consisted of two primary sections. The first section collected general information, including demographics, training habits, pool behavior, and general skin health before and after taking up swimming. The second section focused on specific details related to various skin infections. Explanations of certain diseases and skin conditions were included in the questionnaire, based on the misunderstandings identified during the pilot study. For the purposes of this study, data were extracted from selected sections of the questionnaires, focusing on demographic characteristics, training practices, timing of infections, and the seasons during which swimmers might be more vulnerable to infections. Additional information was gathered regarding the type of swimming facility, years of training, daily training hours, swimming-related behaviors, and history of virus infections and skin allergies. Participants with recurrent infections were asked to provide details about their most recent episode, as recent events are generally recalled more accurately by respondents (Supplementary S1).

### Statistical Analysis

Categorical variables were presented as absolute numbers (*n*) and related frequencies (%). The chi-square test was used to assess association between two categorical variables. To explore relationships between a categorical variable and an ordinal variable, the chi-square trend test was applied. Bivariate correlations were analyzed between superficial bacterial skin infections and factors such as “gender,” “type of facility,” “years of training,” “weekly training” and “daily training hours”. Correlations were examined between the superficial bacterial skin infections and swimmer behaviors, including “walking barefoot on the pool deck” and “sharing equipment.”

Multivariate logistic regression analyses were applied to investigate the relationship between bacterial infections and behavioral factors in the pool area, as well as equipment use. Specifically, for folliculitis, the use of fins and puddles was assessed. For impetigo, “placing bathrobes or clothes on the pool bench” and the use of equipment such as fins, puddles, kickboards, and flip-flops were evaluated. For pitted keratolysis, “placing bathrobes or clothes on the pool bench” and the use of equipment including fins, puddles, and kickboards were examined. Odds ratios (ORs) and 95% confidence intervals (CIs) were calculated for each model. Statistical significance was set at 0.05 to ensure transparency and facilitate interpretation of the results. Data analysis was performed using IBM SPSS 26.0 software (Statistical Package for Social Sciences) (IBM, Armonk, NY, USA).

## 3. Results

### 3.1. Demographic Characteristics

A total of 1047 swimmers participated in the study (response rate: 9.23%), including 577 females (55.1%) and 470 males (44.9%). Most participants trained at outdoor facilities (*n* = 637, 60.8%), while 470 (39.2%) used indoor pools. The largest age group was 9–12 years (*n* = 359, 34.3%), followed by 13–14 years (*n* = 231, 22%), 15–16 years (*n* = 194, 18.6%), 17–18 years (*n* = 112, 10.6%), and over 18 (*n* = 151, 14.4%). Training experience ranged mostly between 7 and 9 years (*n* = 265, 25.3%) and 4 and 6 years (*n* = 262, 25%). About half of the swimmers (*n* = 541) followed a daily training routine of two hours, according to responses from both athletes and their parents.

### 3.2. General Characteristics of Superficial Bacterial Skin Infections and Their Association with Training Routines

#### 3.2.1. Folliculitis

Folliculitis was reported by 28 swimmers (2.7%), with two-thirds experiencing it only once. The most commonly affected areas were the lower limbs (*n* = 12, 42.8%) and torso (*n* = 8, 28.6%). Half of the cases occurred in spring (*n* = 14), while winter (*n* = 6, 21%), summer (*n* = 6, 21%) and autumn (*n* = 8, 7.2%) showed lower frequencies. Most swimmers (*n* = 20, 71.4%) continued training during treatment, whereas 8 suspended training temporarily. A dermatologist was consulted by 57.1% (*n* = 16), while 32.1% (*n* = 90 managed symptoms without medical help (Table 1). Prevalence was higher in females (*n* = 18, 3.1%) than males (*n* = 10, 2.1%) and significantly greater in adult and long-term swimmers (*n* = 15, 7.4%, *p* < 0.001) (Table 2). No associations were found with training frequency, daily hours, or facility type.

#### 3.2.2. Impetigo

Impetigo was reported by 114 swimmers (10.9%). Most cases (68.4%, *n* = 78) occurred once, while 19.3% (*n* = 22) reported it twice. The face (30.1%, *n* = 34) and lower limbs (25.3%, *n* = 29) were most affected. Nearly half of the infections (45.7%, *n* = 52) occurred in summer. Training was interrupted for less than a month by 39.3% (*n* = 45), while 14.3% (*n* = 16) continued training. Dermatological consultation and treatment were sought by 86% (*n* = 98), and 11.4% (*n* = 13) self-treated (Table 1). No significant sex difference was found. The highest prevalence appeared in juniors, particularly swimmers ≤ 14 years. Among indoor swimmers, 13.2% (*n* = 52) reported impetigo (*p* = 0.057). A significant association was found with daily training duration: those training up to 1.5 h daily had the highest infection rate (13.2%, *n* = 41, *p* < 0.001), correlating with their younger age profile (Table 2).

#### 3.2.3. Pitted Keratolysis

Pitted keratolysis was reported by 33 swimmers (3.2%), most commonly during winter and spring. In 60.6% (*n* = 20) of cases, the condition occurred only once. The majority (75.7%, *n* = 25) continued training, while 54.5% (*n* = 18) consulted a dermatologist and received treatment. A smaller proportion (11.4%, *n* = 13) self-managed the infection (Table 1). Prevalence was 4% (*n* = 10) among male and 2.1% (*n* = 23) among female swimmers. Significant associations were found with age (*p* = 0.003) and years of swimming experience (*p* < 0.001), with the highest rates in adults and swimmers over 12 years old (Table 2).

### 3.3. Correlation Between Swimmers’ Behavior and Habits and the Development of Superficial Bacterial Skin Infections

#### Correlation of Superficial Bacterial Skin Infections and Swimmers’ Behavior and Habits

Statistical analysis was conducted to explore the connection between superficial bacterial skin infections and behavioral factors in the pool area. Folliculitis was significantly associated with sharing swimming equipment, particularly fins (*p* = 0.001) and puddles (*p* = 0.006). Further analysis showed that sharing fins increased the risk of folliculitis (OR 2.611, 95% CI: 1.141–5.973, *p* = 0.023) (Table 3). Regarding impetigo, 89 swimmers (11.7%) reported walking barefoot in the pool area, while 90 (13.1%, *p* = 0.001) placed personal clothing on pool benches. Sharing of fins (*p* = 0.012), kickboards (*p* = 0.006), and flip-flops (*p* = 0.026) was also associated with impetigo. The odds of developing impetigo doubled for those placing clothing on benches (OR 2.097, CI 1.301–3.379, *p* = 0.002), sharing kickboards (OR 1.894, CI 1.173–3.059, *p* = 0.009), and flip-flops (OR 2.109, CI 1.306–3.406, *p* = 0.002). For pitted keratolysis, 25 of the 33 affected swimmers reported walking barefoot on pool decks. Significant associations were also found with placing clothing on benches (*p* = 0.036) and sharing fins (*p* = 0.007) and puddles (*p* = 0.003) (Table 3).

## 4. Discussion

The growing number of athletes has contributed to an increase in superficial bacterial skin infections [1], some of which are related to swimming pool conditions and swimmers’ personal habits. Others may be influenced by external, non-sport-specific factors. However, research on skin health in competitive swimmers remains scarce, limiting comparisons across studies. The existing literature mostly reports fungal, bacterial, viral infections, and contact dermatitis as common sports-related skin conditions [10,18]. To our knowledge, this is the first study to investigate the epidemiology of superficial bacterial infections in a large cohort of competitive swimmers, as previous studies have been restricted to case reports and reviews.

This study found that 2.7% of competitive swimmers reported experiencing folliculitis. The overall prevalence in the general population has not been established due to the heterogeneity of folliculitis subtypes [19]. However, one study reported a prevalence of 1.3% among schoolchildren [20], while another documented a rate of 27% in immunosuppressed transplant recipients [21]. A UK clinical study noted a doubling of cases over a decade [22]. Lin et al. [7] observed that previous studies included more males, whereas in our study, cases were slightly higher among females—possibly due to more frequent hair removal—though gender was not statistically associated with prevalence. Folliculitis was more common among adults. Hot tubs account for over 80% of cases in athletes, while pools represent about 20% [10]. In contrast to previous studies reporting peak incidence in autumn [23,24], our findings demonstrated a higher frequency in spring, corresponding with the onset of the competitive season and the rise in shaving practices for both performance and esthetic purposes [25].

Bacterial folliculitis typically affects children and adults with risk factors for increased skin colonization [7]. In infants and children, common sites include the face, buttocks, and axillae; in adolescent girls, the legs; and in boys, flexural areas [7]. In our study, the most affected areas—the face and lower limbs—are consistent with these patterns. While Staphylococcus aureus is the most common pathogen, other bacteria such as *Streptococcus*, *Proteus,* and coliforms have been implicated. *Pseudomonas aeruginosa*, commonly present in swimming pools, is associated with waterborne folliculitis, particularly affecting shaved or exposed skin areas [26,27]. Most participants (75%) reported a single episode, possibly linked to temporary contamination. Dermatological treatment may have contributed to low recurrence. *Pseudomonas* thrives in warm, humid conditions, such as on pool decks and benches, and can grow at temperatures up to 41 °C [28]. Although placing clothing on benches showed no significant correlation with infection in our data or the literature, equipment sharing remains a documented risk factor. In swimming pools, moderate water temperatures (appr. 26 °C), together with overcrowding and turbulence, may decrease chlorine concentrations and facilitate bacterial proliferation [29].

In our study, 10.9% of swimmers reported impetigo, a rate within the global prevalence range of 1.9–49%, which varies by region and living conditions [30]. The highest occurrence was noted in the 9–12-year age group (12.8%), consistent with literature indicating peak rates in childhood and a decline with age [31]. This pattern may reflect increased equipment use and sharing in younger swimmers. Most cases were isolated, likely due to effective dermatological treatment and adherence to management protocols. Affected body sites included the face (25.3%), lower limbs (25.3%), and torso (22.9%), similar to findings by Steele et al. [32]. Frequent hand-to-face contact and shared use of kickboards and flip-flops may explain this distribution. Torso and limb involvement, particularly in adolescents aged 13–16, contrasts with prior reports that emphasize facial lesions [10,33]. Impetigo was more common in summer and early fall, aligning with most studies [33,34,35]. Equipment sharing and personal hygiene practices likely influence the seasonal and anatomical patterns observed.

The present study reports a prevalence of pitted keratolysis of 3.2% among competitive swimmers, representing, to our knowledge, the first large-scale epidemiological investigation of this condition in this athletic population. Previous evidence on pitted keratolysis has been largely limited to isolated case reports or localized outbreaks in swimming facilities [36]. The reported prevalence in other populations ranges from 1.5% among industrial workers to 6.3% in field workers, primarily attributed to walking barefoot and exposure to humid environments [37,38]. Within our cohort, the majority of cases occurred during the winter and spring months, a pattern consistent with the observations reported by Sical et al. [39]. These findings contrast with reports from studies in the general population, which describe seasonal peaks during the warmer and rainy months [40].

The condition was more frequently reported among swimmers over the age of 14, aligning with findings indicating highest prevalence between the ages of 21 and 30 [41]. Behavioral factors, including barefoot walking on abrasive, anti-slip pool surfaces, were commonly noted among affected participants (*n* = 25), suggesting a potential contribution of microtrauma and suboptimal hygiene practices. Interestingly, a higher prevalence among female swimmers was observed, diverging from previous findings and possibly reflecting differences in health-seeking behavior [42]. Emphasis on surface maintenance and disinfection practices is essential for infection prevention in aquatic environments.

To our knowledge, this is the first study to investigate the prevalence of folliculitis, impetigo, and pitted keratolysis among competitive swimmers. Owing to COVID-19 restrictions, data collection was conducted via a self-administered online questionnaire without clinical verification, which may have introduced recall bias. To minimize potential misinterpretation, the survey included standardized definitions of the relevant skin conditions. Although the questionnaire addressed a range of dermatological problems, the present analysis focused specifically on superficial bacterial infections. A key limitation of this study is the absence of clinical validation by a dermatologist; all diagnoses were based on self-reported data and participants’ experiences following dermatological consultation. Moreover, the lack of a control group precludes direct assessment of the overall risk of these infections compared with the general population. Nevertheless, within the swimming cohort, individual risk factors could still be explored, acknowledging the caveat regarding diagnostic accuracy. To reduce the likelihood of selection bias, the questionnaire was distributed to the entire target population, and the relatively large number of respondents enhances the representativeness of the sample. However, environmental factors such as pool water quality were not evaluated. Future research should incorporate such variables to enable a more comprehensive assessment of infection risk in aquatic sports.

This study presents new insights into superficial bacterial skin infections among competitive swimmers, highlighting associations with the swimming pool environment. Although swimming equipment and pool areas may harbor bacteria, the precise infection sources remain unclear. Addressing factors such as poor water quality, overcrowding, and inadequate equipment maintenance is crucial to reduce infection risk [43]. Notably, many infected swimmers continued training during treatment, potentially facilitating transmission. Educational programs should emphasize transmission pathways, hygiene, clinical management, and return-to-play guidelines [44]. Preventive measures include showering before swimming, hand hygiene, avoiding training when infected, and using personal equipment [44]. Additionally, swimmers should be advised to wear flip-flops and avoid walking barefoot in shared spaces, with annual dermatological screenings encouraged [45]. Facility operators and governing bodies must enforce hygiene regulations to ensure swimmer safety. Future research should explore infection prevalence and risk factors across different sports disciplines.

## 5. Conclusions

This study highlights findings of particular relevance to Greek competitive swimmers. The high prevalence of the three superficial bacterial skin infections investigated underscores the unique vulnerability of swimmers, likely due to prolonged exposure to moist environments, shared equipment, and hygiene habits. These results emphasize the importance of targeted preventive measures, early diagnosis, and awareness campaigns within the athletic community to mitigate the impact of such infections and safeguard athlete health.

## Figures and Tables

**Table 1:** Superficial bacterial skin infections.

	Folliculitis *n* (%)	Impetigo *n* (%)	Pitted Keratolysis *n* (%)
Yes	28 (2.7)	114 (10.9)	33 (3.2)
No	1019 (97.3)	933 (89.1)	1014 (96.8)
Number of reported infection episodes			
One	21 (75)	78 (68.4)	20 (60.6)
Two	0	22 (19.3)	5 (15.2)
Three	2 (7.1)	11 (9.6)	5 (15.2)
Four	3 (10.7)	0	0
Five	0	1 (0.9)	1 (3)
≥Six	2 (7.1)	2 (1.8)	2 (6.1)
Infection sites			
Face	4 (14.3)	34 (30.1)	0
Torso	8 (28.6)	26 (22.9)	0
Upper limbs	4 (14.3)	25 (21.7)	0
Lower limbs	12 (42.8)	29 (25.3)	33 (100)
Season during which infection occurred *			
Winter	6 (21.4)	28 (24.7)	12 (33.3)
Spring	14 (50)	20 (17.3)	12 (33.3)
Summer	6 (21.4)	52 (45.7)	8 (22.2)
Autumn	2 (7.2)	14 (12.3)	4 (11.1)
Duration of training interruption due to infection			
<1 week	6 (21.4)	33 (28.6)	4 (12.1)
<1 month	2 (7.2)	45 (39.3)	2 (6.1)
>1 month	0	20 (17.8)	2 (6.1)
Non-training interruption	20 (71.4)	16 (14.3)	25 (75.7)
Type of diagnosis and treatment received			
Dermatologist consultation and treatment	16 (57.2)	98 (86)	18 (54.5)
Only pharmaceutical treatment	9 (32.1)	13 (11.4)	13 (39.4)
None of the above	3 (10.7)	3 (11.4)	2 (6.1)

* More than one answer.

**Table 2:** Bivariate analysis using folliculitis, impetigo, and pitted keratolysis as dependent variables.

	Folliculitis *n* (%)			Impetigo *n* (%)			Pitted Keratolysis *n* (%)		
Characteristic	Yes	No	*p* Value	Yes	No	*p* Value	Yes	No	*p* Value
Gender			0.322 ^a^			0.450 ^a^			0.087 ^a^
Male	10 (2.1)	460 (97.9)		47 (10)	423 (90)		10 (2.1)	460 (97.9)	
Female	18 (3.1)	559 (96.9)		67 (11.6)	510 (88.4)		23 (4)	554 (96)	
Swimming categories			<0.001 ^b^			0.024 ^b^			0.003 ^b^
Junior									
9–12 years old	3 (0.8)	356 (99.2)		46 (12.8)	313 (87.2)		1 (0.3)	358 (99.7)	
Age groups									
13–14 years old	5 (2.2)	226 (97.8)		28 (12.1)	203 (87.9)		7 (3)	224 (97)	
15–16 years old	2 (1.1)	192 (98.9)		21 (10.8)	173 (89.2)		9 (4.7)	185 (95.3)	
17–18 years old	2 (1.8)	110 (98.2)		6 (6.4)	106 (94.6)		6 (6.4)	106 (94.6)	
Men–Women	16 (10.6)	135 (89.4)		13 (8.6)	138 (91.4)		10 (6.6)	141 (93.4)	
Type of swimming facility			0.234 ^a^			0.057 ^a^			0.978 ^a^
Outdoor facility	14 (2.2)	623 (97.8)		60 (9.4)	577 (90.6)		20 (3.1)	617 (96.9)	
Indoor facility	14 (3.4)	396 (96.6)		54 (13.2)	356 (86.8)		13 (3.2)	397 (96.8)	
Years of training			<0.001 ^b^			0.391 ^b^			<0.001 ^b^
≤3 years	1 (1)	99 (99)		14 (14)	86 (86)		1 (1)	99 (99)	
4–6 years	6 (2.3)	256 (97.7)		28 (10.7)	234 (89.3)		2 (0.8)	260 (99.2)	
7–9 years	1 (0.4)	264 (99.6)		31 (11.7)	234 (88.3)		5 (1.9)	260 (99.1))	
10–12 years	5 (2.3)	211 (97.7)		26 (12)	190 (88)		9 (4.2)	207 (95.8)	
>12 years	15 (7.4)	189 (92.6)		15 (7.4)	189 (92.6)		16 (7.8)	188 (92.2)	
Weekly training frequency			0.630 ^b^			<0.001 ^b^			0.088 ^b^
≤3 times	4 (2.7)	145 (97.3)		31 (20.8)	118 (79.2)		6 (4)	143 (96)	
4–5 times	6 (2)	301 (98)		19 (6.2)	288 (93.8)		4 (1.3)	303 (98.7)	
≥6 times	18 (3)	573 (97)		64 (10.8)	527 (89.2)		23 (3.9)	568 (96.1)	
Daily training duration			0.801 ^b^			0.289 ^b^			0.114 ^b^
≤1.5 h/day	8 (2.6)	302 (97.3)		41 (13.2)	269 (86.8)		6 (1.9)	304 (98.1)	
2 h/day	16 (2.9)	527 (97.1)		54 (9.9)	489 (90.1)		23 (4.2)	520 (95.8)	
>2 h/day	4 (2.1)	190 (97.9)		19 (9.8)	175 (90.2)		4 (2.1)	190 (97.9)	

Values are expressed as *n* (%), unless stated otherwise. ^a^ X^2^ test; ^b^ X^2^ test for trend.

**Table 3:** Correlation between superficial cutaneous bacterial infections and swimmers’ behavior and habits.

	Folliculitis *n* (%)		Impetigo *n* (%)		Pitted Keratolysis *n* (%)	
	Yes	No	Yes	No	Yes	No
Walking barefoot on the pool deck						
	*p* = 0.465		*p* = 0.158		*p* = 0.670	
Yes	22 (2.9)	737 (97.1)	89 (11.7)	670 (88.3)	25 (3.3)	734 (96.7)
No	6 (2.1)	282 (97.9)	25 (8.7)	263 (91.3)	8 (2.8)	280 (97.2)
Placing bathrobes or clothing on the pool bench						
	*p* = 0.098		*p* = 0.001		*p* = 0.036	
Yes	12 (2)	596 (98)	90 (13.1)	596 (86.9)	25 (4.1)	583 (95.9)
No	16 (3.6)	423 (96.4)	24 (6.6)	337 (93.4)	8 (1.8)	431 (98.2)
Sharing swimming equipment						
Fins	*p* = 0.001		*p* = 0.012		*p* = 0.007	
Yes	19 (4.7)	382 (95.3)	56 (14)	345 (86)	20 (5)	381 (95)
No	9 (1.4)	637 (98.6)	58 (9)	588 (91)	13 (2)	633 (98)
Puddles	*p* = 0.006		*p* = 0.057		*p* = 0.003	
Yes	18 (4.4)	392 (95.6)	54 (13.2)	356 (86.8)	21 (5.1)	389 (94.9)
No	10 (1.6)	627 (98.4)	60 (9.4)	577 (90.6)	12 (1.9)	625 (98.1)
Kick board	*p* = 0.206		*p* = 0.006		*p* = 0.075	
Yes	22 (3.1)	685 (96.9)	90 (12.7)	617 (87.3)	27 (3.8)	680 (96.2)
No	6 (1.8)	334 (98.2)	24 (7.1)	316 (92.9)	6 (1.8)	334 (98.2)
Flip-flops	*p* = 0.260		*p* = 0.026		*p* = 0.300	
Yes	8 (3.8)	203 (96.2)	32 (15.2)	179 (84.8)	9 (4.3)	202 (95.7)
No	20 (2.4)	816 (97.6)	82 (9.8)	754 (90.2)	24 (2.9)	812 (97.1)

## Data Availability

Data are contained within the article.

## Supplementary Materials

The following supporting information can be downloaded at: https://www.mdpi.com/article/10.3390/idr17050133/s1, S1: Questionnaire.
