# Supplementary material for: Common Superficial Bacterial Skin Infections Self-Reported by 1047 Greek Competitive Swimmers: A 2021 Retrospective Study
Source: Infect Dis Rep. 2025 Oct 20;17(5):133. doi: 10.3390/idr17050133 (PMC12564129; doi:10.3390/idr17050133)
Supplement: Supplementary file 1 [file idr-17-00133-s001.zip › idr-3795720-supplementary.pdf]

VOLUNTEER CONSENT FORM FOR PARTICIPATION IN RESEARCH

Coordinating Institution: University of West Attica, Department of Biomedical Sciences

Ethics Approval: Ethics Committee by University of West Attica (52645-20 July 2020)

Approved by the Hellenic Swimming Federation (787/15-03-2019)

Title: Epidemiological Study of Dermatological Diseases in Competitive Swimmers

Purpose of the Study:

This research aims to examine the prevalence of skin conditions among competitive swimmers. This nationwide study represents a groundbreaking initiative in documenting dermatological issues faced by these athletes. It will furnish the Hellenic Swimming Federation (KOE), which includes its teams and athletes, with crucial insights into swimmer safety concerning cutaneous infectious diseases as well as environmental factors such as water quality, temperature, sunlight, and wind. The findings will help identify the most prevalent skin conditions among Greek swimmers, investigate their potential causes, and recommend suitable protective strategies.

The study focuses on swimmers aged 9 to 12 years old, age group categories (+13 years old) and adult swimmers.

Main Investigator's Full Name: Eleni Sfyri, Lecturer

Contact Information: Email: elsfiri@uniwa.gr

Consent:

I provide my consent to participate in this research study, either for myself or on behalf of my child.

I acknowledge that the data collected may be utilized in conference presentations and/or published in scientific journals.

Participation is voluntary, and I am not obligated to answer every question; I also have the right to withdraw from the study at any time, even after signing this consent form.

The anonymity of my participation or that of my child will be preserved.

I have reviewed the information provided above and consent to participate in the study, either personally or on behalf of my child.

NOTE

We invite you to participate in a research about cutaneous infections in Greek Competitive Swimmers. This research is carried out with the approval of the Swimming Federation of Greece (documents 787/e – 15/3/2019, 2275/e - 22/6/2020) and in the framework of its cooperation with the University of West Attica / School of Health and Welfare Sciences / Department of Biomedical Sciences. The Ethics committee of research of the University of West Attica has, also, approved this research (no. 48855/8.7.2020).

The research concerns swimmer's junior category, 9 to 12 years old, age group categories (13-14, 15-16, 17-18) and Men Women. The questionnaire will be completed by parents/guardians if the swimmers is underage.

- Participation is strictly anonymous.
- In case you have more than one child - swimmer in these categories please repeat the procedure.
- Completing the questionnaire takes about 15 minutes.

The research is headed by Mrs. Sfyri Eleni, Lecturer in Applications - Department of Biomedical Sciences, University of West Attica, Bachelor in Physical Education/Swimming.

E mail address: elsfiri@uniwa.gr

You must consent if you want to participate to this study.

Consent ☐ No consent ☐

Part A

In Part A you note with an X your answer in the last column where required.

Symbol * means that the answer is mandatory

1.	*Gender	Male	
		Female	
2.	*Swimmer's age category	9 – 12 years old	
		13 – 14 years old	
		15 – 16 years old	
		17 – 18 years old	
		Older than 18 years old	
3.	Swimming club (optionally)	…………………………………………………………	
4.	*County of the swimming pool facility	…………………………………………………………	
5.	*Type of swimming pool facility	Outdoor facility	
		Indoor facility	
6.	*Training years	Less/equal than 3 years	
		4 – 6 years	
		7 – 9 years	
		10 – 12 years	
		More than 12 years	
7.	*Weekly training	Less/equal than 3 times	
		4 – 5 times	
		More than 6 times	
8.	*Number of hours of daily training in the water	Less/equal than 1.5 hours/day	
		2 hours/day	
		More than 2 hours/day	
9.	*Behavior in the swimming pool facility (more than one options):	Wearing flip flops	
		Walking barefoot on the pool's deck	
		Placing clothes/bathrobes on the pool's bench	
10.	*Sharing equipment (more than one options)	Training fins	
		Hand paddles	
		Kickboard	
		Flip flops	
		None of the above	
11.	*Occurrence of allergies (more than one options)	Seasonal allergy (nasal discharge and skin manifestations that appeared during specific times of the year)	
		Respiratory allergy (nasal discharge and congestion caused by environmental factors)	
		Skin allergy (contact and/or atopic dermatitis)	
12.	*History of skin manifestations before taking up swimming	Yes	
		No	

13.	Skin manifestations occurring during years of swimming training	Yes	
		No	

PART B.

In this part of the questionnaire, answer the questions about the following skin infections you have experienced as many years as you swim.

- in C, D, E, F, G questions report only on the last time of infections.

**A. CUTANEOUS MANIFESTATIONS**		
Bacterial infections	Explanation	Yes
Folliculitis	It is an inflammation of the hair follicles, appearing as bumps or a rash on the skin in areas where hair grows, such as the scalp, torso, arms, and legs	
Impetigo	Impetigo presents as sores and blisters around the mouth and nose. It can also appear on the scalp, legs, and arms. It is commonly caused by Staphylococcus bacteria.	
Pitted Keratolysis	Infection that most commonly affecting the soles, it is characterized by small, crater-like pits and a foul odor	
Virus infections		
Warts	Common warts are small, rough bumps on the skin that usually appear on the fingers or hands and often have tiny black dots	
Molluscum Contagiosum	A common skin infection that causes raised, round, skin-colored bumps with a small indentation or dot in the center.	

SKIN INFECTIONS	B. NUMBER OF INFECTIOUS EVENTS*					
	One	Two	Three	four	Five	≥six
Folliculitis						
Impetigo						
Pitted Keratolysis						
Warts						
Molluscum Contagiosum						

	C. SITES OF INFECTIONS (more than an answer)*				
	Face	Torso	Upper limbs	Lower limbs	Soles
Folliculitis					
Impetigo					
Pitted Keratolysis					
Warts					
Molluscum Contagiosum					

	D. INTERRUPTION PERIOD OF THE TRAINING PROGRAM DURING TREATMENT OF THE SKIN INFECTION*			
	No training interruption	Less than a week	Less than a month	More than six months
Folliculitis				
Impetigo				
Pitted Keratolysis				
Warts				
Molluscum Contagiosum				

	E. SEASON OF SKIN INFECTIONS (more than an answer)*			
	Winter	Spring	Summer	Autumn
Folliculitis				
Impetigo				

Pitted Keratolysis				
Warts				
Molluscum Contagiosum				

	**F. DERMATOLOGIST EXAMINATION FOR CERTAIN SKIN INFECTIONS ***		**G. TREATMENT FOR CERTAIN SKIN INFECTIONS***	
	**Yes**	**No**	**Yes**	**No**
Folliculitis				
Impetigo				
Pitted Keratolysis				
Warts				
Molluscum Contagiosum				

Thank you for your participation!
